# Identification of genetic determinants of bedaquiline resistance in *Mycobacterium tuberculosis* in Ural region, Russia

**DOI:** 10.1128/spectrum.03749-23

**Published:** 2024-02-12

**Authors:** Tatiana Umpeleva, Elena Chetverikova, Danila Belyaev, Natalya Eremeeva, Tatiana Boteva, Ludmila Golubeva, Diana Vakhrusheva, Irina Vasilieva

**Affiliations:** 1 Department of Microbiology and Preclinical Research, National Medical Research Center of Phthisiopulmonology and Infection Disease, Yekaterinburg, Russia; 2 Laboratory of Medical Chemistry, Postovsky Institute of Organic Synthesis, Ural Branch of the Russian Academy of Sciences, Yekaterinburg, Russia; 3 Administration, National Medical Research Center of Phthisiopulmonology and Infection Disease, Moscow, Russia; Foundation for Innovative New Diagnostics, Geneva, Switzerland

**Keywords:** *M. tuberculosis*, mutations, bedaquiline, nanopore sequencing, drug susceptibility testing

## Abstract

Collecting data on rare *Mycobacterium tuberculosis* (*Mtb*) clinical isolates with resistance to the new anti-tuberculosis drug bedaquiline is an important task for improving antimicrobial susceptibility testing methods. Nanopore whole genome sequencing, the proportion method on Middlebrook 7H11 medium, and BACTEC MGIT 960 assays were used to analyze genotypic and phenotypic resistance to bedaquiline. We found four mutations: *atpE* I66M, *atpE* А63Р, *Rv0678* А36Т, and *Rv0678* S53P in five isolates with different levels of phenotypic bedaquiline resistance.

**IMPORTANCE:**

Bedaquiline (BDQ) is a new anti-tuberculosis drug. The phenotypic and genotypic data describing the mechanism of drug resistance are critical for the design of rapid and accurate diagnostic tests. We consider that our work, which describes genotypic and phenotypic resistance to BDQ, can contribute to the standardization of drug susceptibility testing.

## INTRODUCTION

World Health Organization (WHO) has classified the new anti-tuberculosis drug bedaquiline (BDQ) as a group A drug for the treatment of multidrug-resistant tuberculosis (MDR-TB). However, since the introduction of BDQ to treatment regimens, *Mtbs* resistance to it has emerged (1).

BDQ is an adenosine triphosphatase synthase inhibitor (2). Mutations in *atpE* (*Rv1305*) gene encoding a transmembrane subunit of the ATP synthase are associated with BDQ resistance (3).

Mutations in *Rv0678*, encoding a transcriptional repressor of the MmpS5-MmpL5 efflux pump, have been shown to be another mechanism of resistance. Highfrequency mutations in the *Rv0678* gene result in low BDQ resistance *in vitro* and are associated with increased cellular efflux of BDQ in clinical settings (4).

Mutations in the putative proline aminopeptidase gene *pepQ* (*Rv3525c*) may cause low-level resistance to BDQ (5). The clinical significance of *pepQ* function and how its loss may result in decreased sensitivity to bedaquiline remain unclear (6).

The WHO catalog of *Mtb* mutations associated with drug resistance (first edition) (7) does not describe any variants in this gene associated with phenotypic resistance to BDQ. Limited data and the fact that some *Rv0678* mutations result in minimum inhibitory concentrations (MICs) close to the critical concentration lead to conflicting categorical phenotypic drug susceptibility testing (pDST) results. In the second edition of catalog (8), six mutations in *Rv0678* were described as associated with resistance and 13 mutations (*Rv0678*, *atpE*, and *pepQ*) were described as associated with resistance–interim.

In this study, we investigated mutations associated with BDQ resistance in phenotypic-resistant *Mtb* isolates using whole genome sequencing (WGS) with Nanopore technology.

## MATERIALS AND METHODS

During the period from November 2019 to December 2021, 239 *Mtb* isolates were obtained from various clinical samples from 203 TB patients who were treated at the Ural Research Institute for Phthisiopulmonology, a branch of the National Medical Research Center of Phthisiopulmonology and Infection Diseases.

pDST to rifampicin (1 mg/L), isoniazid (0.1 mg/L), ethambutol (5 mg/L), levofloxacin (1 mg/L), moxifloxacin (0.5 mg/L), and amikacin (2 mg/L) was performed using agar proportion method on Middlebrook 7H10 medium (Becton Dickinson and Company, USA) and to bedaquiline (1 mg/L) and delamanid (0.06 mg/L) using BACTEC MGIT 960 technology (Becton Dickinson and Company, USA) (9). Resistance to both isoniazid and rifampicin was defined as multidrug resistance (MDR). *Mtb* suspension aliquots prepared for pDST for all isolates were stored at −80°C in 20% glycerol. For all BDQ-resistant isolates, a fresh aliquot of the stored cells was thawed and sub-cultured in Lowenstein-Jensen medium (Himedia, India) for DNA extraction for whole genome sequencing and determination of BDQ MICs.

For these isolates, the MIC of BDQ was determined by the serial dilutions’ method on Middlebrook 7H11 medium (Becton Dickinson and Company, USA) (0.12, 0.25, 0.5, 1, 2, 4, 8, and 16 mg/L), critical concentration was 0.25 mg/L as well as using BACTEC MGIT 960 technology (Becton Dickinson and Company, USA) (0.5, 1, 2, 4, 8, 16, and 32 mg/L) critical concentration was 1 mg/L. The virulent laboratory strain *Mtb* H37Rv (TMC#102, FSBI Tarasevich State Control Institute, Moscow, Russia) was used as a control strain for pDST.

Genomic DNA (gDNA) was extracted from aliquots of *Mtb* suspension prepared for DST using cetyltrimethylammonium bromide (CTAB) method (10). One nanogram of input gDNA was used for library preparation. For genomic DNA library preparation, all steps were performed according to the gDNA Ligation sequencing protocol—native barcoding (SQK-LSK109 with EXP-NBD04) (Oxford Nanopore Technologies, Oxford, UK). WGS was performed on MinION platform with R9 flow cells. Base calling was done using Guppy 6.2.1 basecaller.

Galaxy platform (https://usegalaxy.eu/) was used for the bioinformatics analysis. Reads were mapped to the Mtb H37Rv reference genome (NC_000962.3) using minimap2 v.2.24 (11). The quality of mapped reads was checked using Qualimap v.2.2.2c (12). TB-profiler v.4.4.1 was used to search for drug resistance mutations and lineage determination (13, 14). We screened for variants in genes known to confer resistance to BDQ including *Rv0678*, *atpE*, and *pepQ*, using Unopro UGENE v.45.0 and Mycobacterial browser (https://mycobrowser.epfl.ch/).

## RESULTS AND DISCUSSION

According to drug susceptibility testing results, 206 (86.2%) of the 239 isolates studied were MDR, including five (2.1%) with resistance to BDQ. WGS was performed on these five isolates, and the MIC of bedaquiline was determined by the proportion method on Middlebrook 7H11 medium, as well as using BACTEC MGIT 960 technology.

Sequencing on the Oxford Nanopore platform yielded median coverage depths ranging from 39 to 298. Mutations of the *atpE* gene were found in three isolates: I66M mutation was identified in two isolates with an MIC of 0.5 mg/L on Middlebrook 7H11 medium (resistant) and 4–8 mg/L on BACTEC MGIT 960 (resistant), an isolate with the А63Р mutation had an MIC of 2 mg/L on Middlebrook 7H11 medium (resistant) and 32 mg/L on BACTEC MGIT 960 (resistant) (Table 1).

**TABLE 1:** The genetic and phenotype data of *Mycobacterium tuberculosis* clinical isolates resistant to bedaquiline^*a*^

Isolate ID	BDQ contained treatment, month	HIV status	BDQ MIC (mg/L) agar proportion method	BDQ MIC (mg/L) BACTEC MGIT 960	Mutations to BDQ			Lineage	Resistance mutations to other anti-TB drugs									
					*atpE*	*Rv0678*	*pepQ*		S *rpsL*/*rrs*	H *katG/fabG1-inhA*	R *rpoB/rpoC*	E *embB*	Pz *pncA*	Fq *gyrA*	Ag rrs/eis	Cs *alr*	Et fabG1-inhA/ethA	PAS folC
25421	36	Pos	2.0	32.0	A63P	wt	wt	2	K43R /517C>T	S315T /15C>T	N435G, L430P	S347I	wt	D94G	/37G>T	wt	15C>T	wt
6721	23	Neg	0.5	8.0	I66M	wt	wt	2	K43R	S315T	S450L/I491T	M306V	V9A	D94N	/10G>A	wt	/1010delT	wt
5521	13	Neg	0.5	4.0	I66M	wt	wt	2	K43R	S315T	S450L	Q497R	wt	A90V	wt	M343T	/327delG	E40G
17221	26	Pos	0.25	8.0	wt	A36T	wt	4	K43R	S315T	S450L/L527V	M306V	Y103 stop	G88A	1401A>G	wt	wt	wt
3320	0	Pos	<0.12	1.0	wt	S53P	wt	2	K43R	S315T/154G>A	D435T	wt	Y103C	D94G	wt	wt	154G>A	wt

^*a*^ S, streptomycin; H, isoniazid; R, rifampicin; Pz, pyrazinamide; Fq, fluoroquinolone; Ag, aminoglycoside; Cs, cycloserine; Et, ethionamide; PAS, para-aminosalicylic acid; BDQ, bedaquiline; and wt, wild type.

According to catalog of mutation, *atpE* I66M and А63Р were determined as associated with resistance–interim (8). Computational analysis of the structural and functional consequences of these variants revealed that resistance-associated variants are primarily localized to the drug-binding site, disrupting key interactions with BDQ and resulting in reduced binding affinity (15, 16). Differences in binding affinity in these sites could be reflected in different MIC values.

Two more isolates had *Rv0678* mutations: А36Т with an MIC of 0.25 mg/L on Middlebrook 7H11 medium (resistant) and 8 mg/L on BACTEC MGIT 960 (resistant). An isolate with S53P mutation had an MIC of less than 0.12 mg/L on Middlebrook 7H11 medium (sensitive) and 1 mg/L on BACTEC MGIT 960 (resistant).

Only *Rv0678* S53P is marked as uncertain significance in catalog of mutation. Mutation *Rv0678* А36Т is not listed in the catalog but previously was described as associated with a bedaquiline-resistant phenotype (low confidence) (17). It is important to note that the *Rv0678* S53P mutation was identified in *Mtb* isolates from BDQ-naive patients, while the other patient had received BDQ for 13–36 months (Table 1). Resistance-conferring mutations in *Rv0678* gene have also been previously reported in BDQ-naive MDR-TB patients (18–20). This finding highlights the importance of genotypic and phenotypic BDQ DST at baseline to ensure appropriate TB treatment.

No *pepQ* gene mutations were detected in all five isolates.

### Conclusion

In this study, we analyzed genotypic and phenotypic resistance to BDQ in *Mtb* clinical isolates obtained from TB patients in Ural region, Russia. Our results contribute to the knowledge of the genetic basis of resistance to BDQ: mutation *Rv0678* А36Т is not listed in the WHO catalog of mutation but previously was described as associated with a bedaquiline-resistant phenotype; *Rv0678* S53P mutation was identified in *Mtb* isolates from BDQ-naive patient. We hope that these results can contribute to the standardization of DST.

## Data Availability

All data generated or analyzed during this study are included in this published article. All genome data were deposited in GenBank (BioProject accession link: https://www.ncbi.nlm.nih.gov/biosample?LinkName=bioproject_biosample_all&from_uid=980952).
